# Osteoproductivity of Injectable Bone Grafts with and without Ostrich Eggshell Membrane Protein in Rabbit Femur

**DOI:** 10.3390/jfb15070201

**Published:** 2024-07-22

**Authors:** Ziya Ozan Cengiz, Ercan Durmus, Ilhami Celik, Ahmet Aktı

**Affiliations:** 1 Oral and Maxillofacial Department, Faculty of Dentistry, Selcuk University, Konya 42250, Turkey; durerc@yahoo.com (E.D.); dt.ahmetakti@gmail.com (A.A.); 2 Department of Histology and Embryology, Faculty of Veterinary medicine, Selcuk University, Konya 42250, Turkey; ilce54@hotmail.com

**Keywords:** bone graft material, dental implant, eggshell powder

## Abstract

Background: The aim of this study was to evaluate the biocompatibility and effectiveness in terms of osseointegration of dental implants composed of novel injectable bone grafts with and without ostrich eggshell particles and membrane protein in rabbit femur. Methods: Sixteen adult male New Zealand rabbits were used in this study. A bone defect was created in each animal’s right and left femur, and a dental implant was placed adjacent to the defect. Two graft materials were prepared, one containing the membrane protein and the other not. In two groups, the defects were filled with these materials. In the negative control group (NC, (n:8)), the defects were left empty. A commercial product of biphasic calcium sulfate was used as a positive control material (PC, *n* = 8). The graft groups were defined as the group with the membrane protein (MP+, (n:8)), and without the membrane protein (MP−, n:8). The animals were euthanized at the 12th week after surgery. The samples were investigated using histology, histomorphometry, and micro-computed tomography. Data were statistically analyzed using one-way ANOVA and Tukey’s tests (*p* = 0.05). Results: Both the PC and MP+ groups had similar newly formed bone areas, and the mean values of these groups were significantly (*p* < 0.05) higher than those of the MP− and NC groups. The PC group had the highest amount of unresorbed material, while the MP− group had the lowest amount of unresorbed material. The bone–implant contact (BIC) scores of the PC and MP+ groups were significantly higher (*p* < 0.05) than that of the NC group. The connective tissue area of the PC group was the lowest, which was significantly lower than the other groups (*p* < 0.05). Conclusions: The grafts produced are highly biocompatible and also showed osteoproductivity. Their cost-effectiveness and osteoproductive activity require further investigation.

## 1. Introduction

Since the pioneering work of Per-Ingvar Brånemark, oral implantology has become a fundamental aspect of modern dentistry. Dental implants significantly improve the treatment options for restoring edentulous patients’ lost function and aesthetics [1]. The long-term success rate of dental implants is estimated to be 96.4% [2,3]. The high success rates of dental implants have increased their application, resulting in many challenging clinical scenarios being presented.

The challenging scenarios are a range of conditions, including periodontal disease, tooth extraction, trauma, pathological conditions, and age-related bone resorption [4]. These conditions reduce horizontal and vertical bone height in the alveolar crests and local defects [5,6]. Furthermore, during dental implant surgery, particularly in horizontally incomplete alveolar crests and alveolar crests with local defects, dehiscence and fenestration-type defects may occur [7]. As a consequence of these defects, the rough surfaces of dental implants may become exposed, which may have a detrimental impact on the survival of the implants. It is recommended that exposed implant surfaces and bone defects be repaired to enhance their long-term success and to prevent aesthetic and functional complications [8].

The use of graft materials of different origins to facilitate bone healing has been extensively documented in the medical literature [9]. The efficacy of these materials as bone substitutes for bone regeneration has been demonstrated to vary to some extent [10,11]. The commercial availability of synthetic bone substitutes for cranio-maxillofacial applications is limited due to the costly manufacturing process. A further reason for the limited availability of data on bone substitutes is the relatively small number of clinical studies conducted to compare them [12]. Consequently, a limited number of bone substitutes currently dominate the material market.

Among the materials studied in the literature, eggshells have been identified as worthy of further investigation regarding their chemical properties [13,14]. Another advantage of OE is that it is a relatively inexpensive material that is readily available and simple to process [15]. Eggshell is a productive bone substitute in treating peri-implant defects, cystic jaw cavities, and interposition grafts [16,17]. This is attributed to its biocompatibility and capacity to bond to the bone site [18]. The composition of OE is strikingly similar to that of mineralized bone matrix, with the majority of the material consisting of calcium carbonate (97.4%), magnesium phosphate (1.9%), and tricalcium phosphate (0.7%) [19]. Furthermore, the calcified eggshell contains an organic matrix that accounts for 2% of the total eggshell weight. The eggshell organic matrix contains a variety of proteins and proteoglycans, including ovocleidin-116, ovotransferrin, ovalbumin, ovocalixin-32, ovocleidin-17, osteopontin, and lysozyme. Some of these can influence calcite crystals’ morphology and precipitation rate during the formation of eggshells [20,21].

In this experimental study, an injectable form of OE and eggshell embrane protein, a material that has demonstrated efficacy when used as a graft, was produced in different formulations to facilitate its clinical use. The effects of these novel materials on the ease of manipulation during application, hardening capacity, osteoconductive properties, bone defect healing, and osseointegration of dental implants were evaluated using clinical, micro-computed tomography (micro-CT), and histomorphometric methods. The null hypothesis is that injectable bone grafts with and without membrane protein produced from OE are not biocompatible and do not enhance osteoproductivity at the implant surface.

## 2. Materials and Methods

### 2.1. Ethical Statement

This experimental study was approved by the Ethics Committee of Selçuk University Experimental Medicine Research and Application Centre (12-2021). The recommendations of the National Institutes of Health Guide for the Care and Use of Laboratory Animals were followed in the care of the animals, and Law No. 5199 of the Constitution of the Republic of Turkey and the ARRIVE guidelines 2.0 were followed in the conducting and reporting of the experimental follow-up [22].

### 2.2. Preparation of the OE Particles and OE Membrane Protein

Fresh ostrich eggs obtained from a commercial source were cracked, eviscerated, and disinfected by soaking the shell pieces with their membranes in 10% sodium hypochlorite for 12 h. The pieces were then washed three times with sterile distilled water for two hours each, dried, and the membrane pieces mechanically separated from the shell pieces. Both eggshell and membrane fragments were oven-dried at 50 °C overnight. The materials were stored in a deep freezer until use.

The water-soluble shell membrane protein was prepared by treating the powdered membrane in 3 parts 1.5 N NaOH and 1 part absolute ethanol mixture (*v*/*v*) at 50 °C for 3 h. When dissolution was completed, the liquid phase was evaporated. The remaining solid fraction was used as the membrane protein. This fraction was washed with absolute methanol and dried overnight at 50 °C under a 300 mbar vacuum. The prepared product was stored in a deep freezer until use.

Two injectable bone cement formulas were prepared, one containing the eggshell membrane protein and the other without the protein. The formula of the eggshell membrane protein-containing cement is given in Table 1.

**Figure 1:**
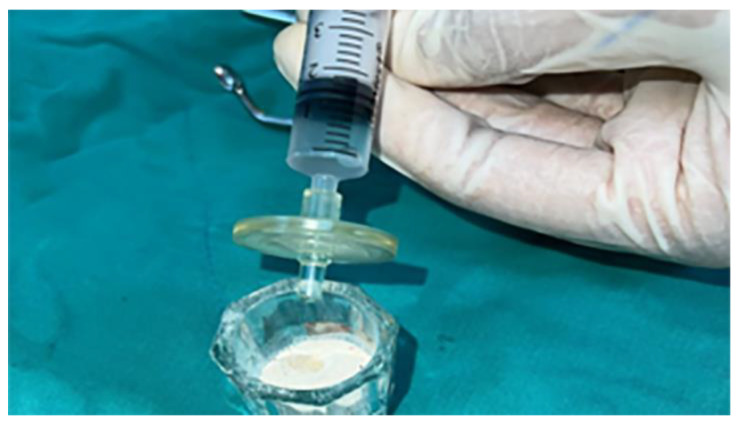
Mixing powder and membrane-filtered liquid was employed to obtain injectable graft material.

The cement formula without the eggshell membrane protein was also prepared as shown in Table 1, without including the eggshell membrane protein. Both mixtures were sterilized with ehhylene oxide. To obtain the cement material during the operation, 4% Na_2_HPO_4_ was included in the mixture until an appropriate consistency was obtained (Figure 1). The mixture was thoroughly mixed and sterilized with ethylene oxide. The powdered mixture was stored in a deep freezer until use.

### 2.3. Experimental Animals

This experimental study used 16 New Zealand white male rabbits aged 10–12 months and weighing 3.7 kg ± 0.15. The Experimental Medicine Research and Application Centre of Selçuk University provided the rabbits. The animals were housed in individually ventilated cages under a 12 h light/dark cycle, with ad libitum access to a standardized diet and filtered water. The animals were randomly allocated to the study groups described below. The welfare of the animals was monitored every week throughout the study period, with no significant issues identified.

### 2.4. Study Design

**Figure 2:**
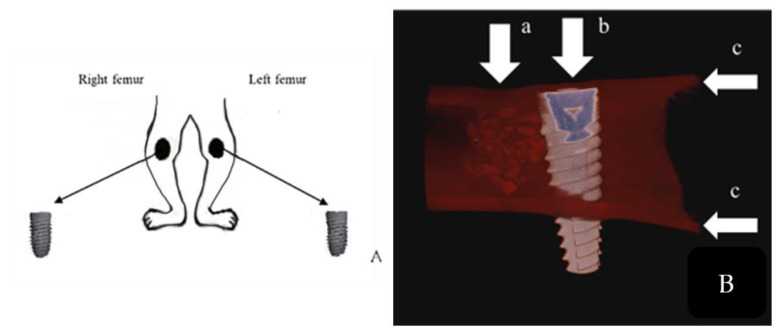
(**A**) The region where each animal’s surgical procedure was performed is indicated. Consequently, eight samples were obtained for each group, with four animals in each group. (**B**) Study design is shown. (a) Defect created and filled with graft material. (b) Bi-cortically placed implant. (c) Cortical bone.

This study was designed as a randomized parallel-group study. The 16 rabbits used in the study were randomly selected and divided into four groups of four animals each. Implants (BIODENTOSS^®^, DOME, Konya, Turkey) were placed in the distal medial parts of the right and left femurs of all animals in each group, and eight specimens were obtained for each group (Figure 2). The diameter of the implants was 3.5 mm, and the length was 11.5 mm. A 4 mm diameter defect was created in the distal medial part of the animals’ femurs using a trephine bur. An implant site was then prepared adjacent to the 4 mm diameter defect, and the implant was placed. After the procedure, the defects were filled with Bond Apatite^®^ in the positive control (PC, *n* = 8), with injectable OE powder and membrane protein (MP+, *n* = 8) in the second group, and with injectable OE powder without membrane protein (MP−, *n* = 8) in the third group. The fourth group was a negative control (NC, *n* = 8) without bone grafting. After the defects were filled, all defects were sealed with a commercial barrier membrane (Maggi^®^. Andezeno, Italy) to ensure standardization.

To ensure randomization, all animal legs were numbered from 1 to 32. Initially, 8 leg numbers were selected and recorded for the NC group using an online random number generator. Subsequently, the same method was applied to the other groups, and the leg numbers for each group were determined and recorded. All procedures were conducted according to these assigned numbers.

### 2.5. Surgical Procedure

All animals were anesthetized by intramuscular injection (0.59 mL/kg) of a combination of ketamine hydrochloride (Ketanes^®^, Alke, Istanbul, Turkey) and xylazine (Rompun^®^, Bayer, Leverkusen, Germany). The surgical procedure was conducted in a sterile environment. A scalpel was employed to incise the subcutaneous fascia on the medial aspect of the femur in a line parallel to the skin. During the procedure, the integrity of the vessels and nerves in the area was maintained. Subsequently, the underlying muscles were incised bluntly to reach the periosteum. The periosteum was then incised and excised. Subsequently, a trephine burr with a diameter of 4 mm was employed to create a defect by cooling with saline, thus exposing the medullary canal. Subsequently, the implant was prepared for the implantation and repairing of any defects. The implant sockets were prepared by cooling with saline solution, after which the implant was placed.

**Figure 3:**
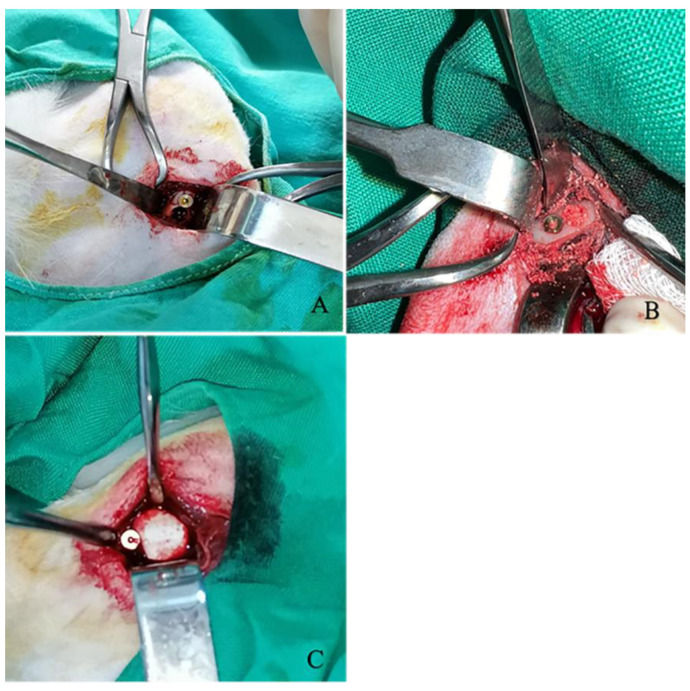
(**A**) The creation of a 4 mm diameter defect and subsequent implant placement adjacent to the defect. (**B**) Filling-in of the defects is shown by the relevant group. (**C**) Closure of all defects with a membrane is shown.

Subsequently, 32 defects were treated with graft applications according to the respective groups and closed with a membrane (Figure 3). Once hemostasis had been achieved, the periosteum and muscles were closed with a 3/0 absorbable multifilament suture following standard surgical procedures. Subsequently, the skin was closed with 3/0 non-absorbable multiflament suture thread. Following the surgical procedure, all animals received intramuscular injections (0.5 mL/kg). The antibiotic Procillin (penicillin G procaine 400,000 IU/mL, Fako, Istanbul, Turkey) was administered. After the surgical procedure, subjects were given ad libitum access to standard rabbit feed and water. The dressings were changed daily, and the healing status of the wounds was observed and recorded. The skin sutures were removed on the tenth day.

At the 12th week postoperatively, the animals were first weighed and then euthanized by intraperitoneal overdose (100 mg/kg) of pentobarbital (Nembutal, 100 mg/mL, Abbott Laboratories, Chicago, IL, USA). The femur specimens, including the implant and defect sites, were dissected and placed in a 10% buffered formalin (0.1 M, pH 7.4) solution.

### 2.6. Micro-Computerized Tomographic (µ-CT) Evaluation

The specimens were subjected to a µ-CT examination using a SkyScan1275 device (serial number 11M01180, Aartselaar, Belgium) at Ankara University, Faculty of Dentistry. The examination was conducted with the software package version 1.7, with a pixel size of 75 µm, a current of 100 µA, and a voltage of 100 kV. A total of 3600 rotations were performed around each implant, resulting in the acquisition of 2000 × 2000 pixel cross-sectional images. The acquired data were recorded using a high-resolution DEXELA_1512 camera (SkyScan^®^, Kontich, Belgium). The recorded data were then analyzed using the SkyScan CT CTAn v1.1.18 Analyser volumetric analysis software (Bruker^®^, Kontich, Belgium). The bone–implant contact (BIC) area was calculated and expressed as a percentage of the total implant area in direct contact with bone tissue.

### 2.7. Histological Investigations and Histomorphometry

The tissue samples were decalcified in 10% ethylenediamine tetraacetic acid (EDTA) at +4 °C for 3 months. After decalcification, the samples were processed by routine histological techniques and immersed in paraffin blocks. From the blocks, 6 µm sections were taken on poly-L-lysine-coated slides using a microtome (SM 2000R, Leica Microsystems, Heidelberg, Germany) and dried at 37 °C overnight. The sections were stained with hematoxylen-eosine, toluidine blue, Crossmon’s trichrome, and Pappenheim’s panoptic staining. Slides were examined under a light microscope (Nikon Eclipse E 400, Nikon Corporation, Chiyodaku, Japan), digital images of the required areas were recorded with a digital camera (DS Camera Head DS-5M, DS Camera control unit DS-L1), and histological and histomorphometric analyses were performed on these images using histomorphometry software v.2.2 (OsteoMeasure, OsteoMetrics, Inc., Decatur, GA, USA). Encapsulation, foreign-body reaction, bone modeling, and remodeling were evaluated, and newly formed bone area, adipose tissue, and vascular and connective tissue area in the defect area were determined in all groups. From the data, the percentage value of each parameter was calculated and statistically analyzed.

### 2.8. Statistical Analysis

The SPSS 22 software was used to analyze the data. The Shapiro–Wilk test was used as a normal distribution test. One-way ANOVA and Tukey’s test were used in the analyses. *p* < 0.05 was considered significant.

## 3. Results 

### 3.1. Clinical Results 

No operation-related complications were observed in the animals. No serious allergic, toxic, or graft rejection reactions to the graft materials or implants were observed. The implants in all the groups were successfully osseointegrated.

### 3.2. Micro-Computerized Tomographic (µ-CT) Results

**Figure 4:**
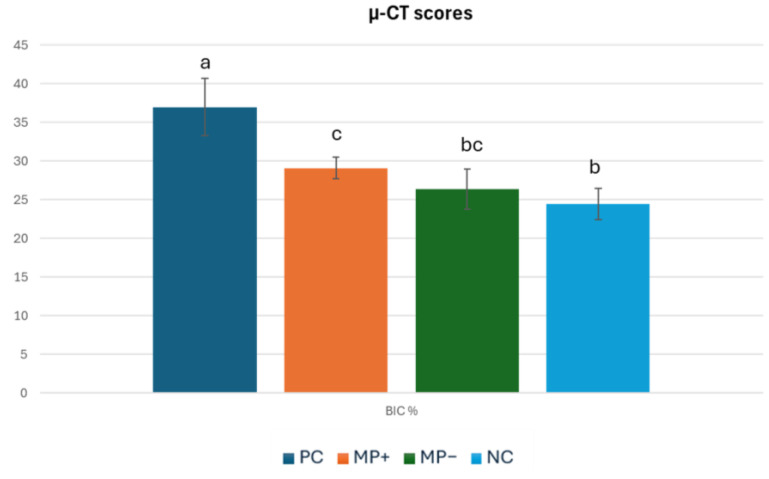
µ-CT scores of the groups. ^a–c^ Different superscripts in each row indicate a statistically significant difference between groups after post hoc analysis.

**Figure 5:**
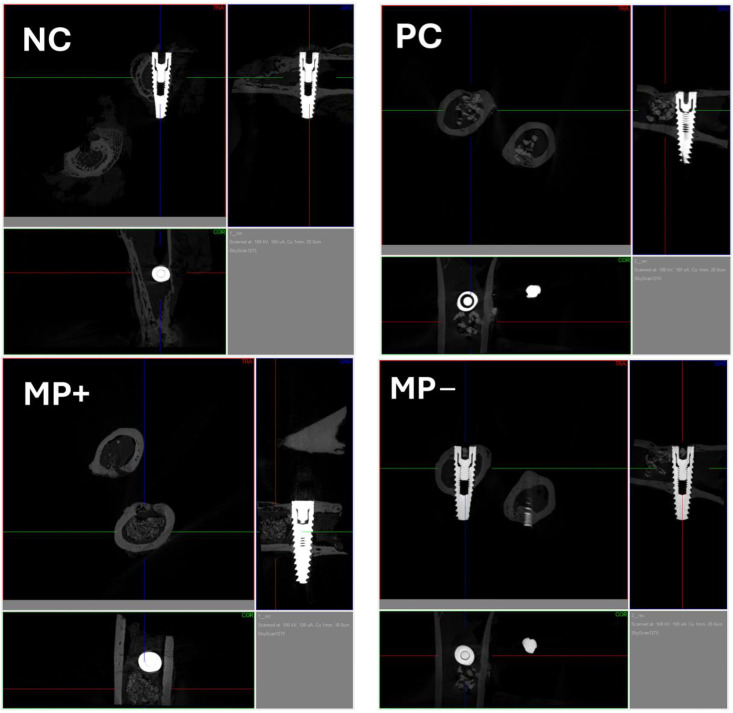
NC: µ-CT of the NC group is shown. PC: µ-CT of the PC group is shown. MP+: µ-CT of the MP+ group is shown. MP−: µ-CT of the MP− group is shown.

The highest BIC value (37.01 ± 3.69%) was found in the PC group, which was placed in the defect area, and this value was significantly (*p* = 0.00) higher than the mean BIC values of the other groups. The lowest BIC value (24.45 ± 2.02%) was found in the NC group where the defect area was left empty. This group was followed by the mean value of MP− (26.4 ± 2.62%), and the mean values of these two groups were very close and the difference was insignificant (*p* = 0.446). The BIC value of the MP+ graft group (29.09 ± 1.44%) was higher than the BIC value of the MP− graft group (26.4 ± 2.62%), but the difference was not significant (*p* = 0.205) (Table 2) (Figure 4) In all the graft groups, bone formation started from the cortical part, and unresorbed graft material was found in the marrow. While unresorbed graft material was observed in the PC group, it was distributed in the connective tissue in the MP+ and MP− groups (Figure 5).

### 3.3. Histological Findings

**Figure 6:**
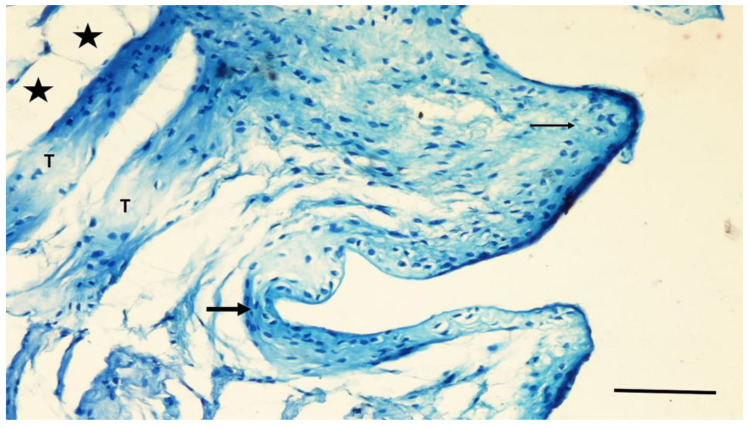
A section from the NC group. Fibrocartilaginous tissue (arrow) covering the grooves and intergroove regions of the screw. Deeper regions are filled with trabecular bone (T) and adipocytes (asterisks). Toluidine blue staining. Magnification bar: 100 µm.

In the NC group, the grooves of the implant in the intercortical region were filled with a thin band of new bone tissue. Fibrocartilaginous tissue was found adjacent to the screw grooves and in the intergroove region (Figure 6). Thin bone trabeculae and myeloid tissue mainly occupied the medullary region associated with the defect site.

**Figure 7:**
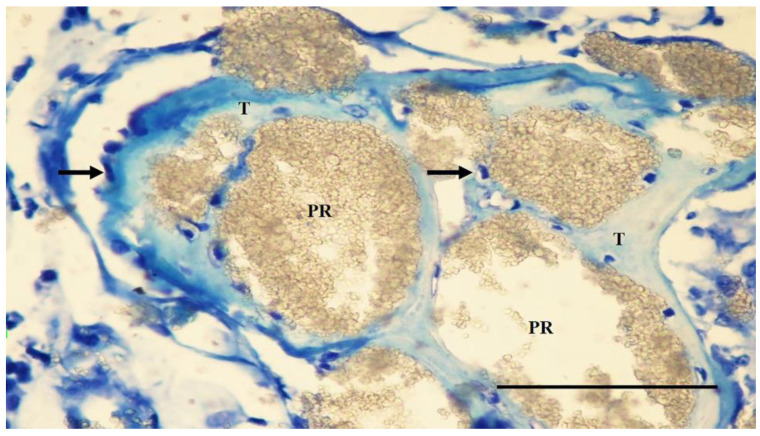
A section through the adjacent defect area of the PC group. Larger Bond Apatite particles (PR) are partially resorbed, while smaller particles are highly resorbed. The particle residues (PR) are surrounded by thin bone trabeculae (T) covered with osteocytes (arrow). Toluidine blue staining. Magnification bar: 100 µm.

In the PC group, the screw bodies in the cortical region were covered with tissue similar to that in the NC group. The graft particles were partially resorbed in the adjacent defect area, while the smaller particles were highly resorbed. Osteoblastic cells covered the surfaces of the primary bone trabeculae surrounding the particles. In some specimens, a weak infiltration of mononuclear cells was seen in the loose connective tissue between the particles. No giant cells or fibrous capsules were observed around the particles (Figure 7).

**Figure 8:**
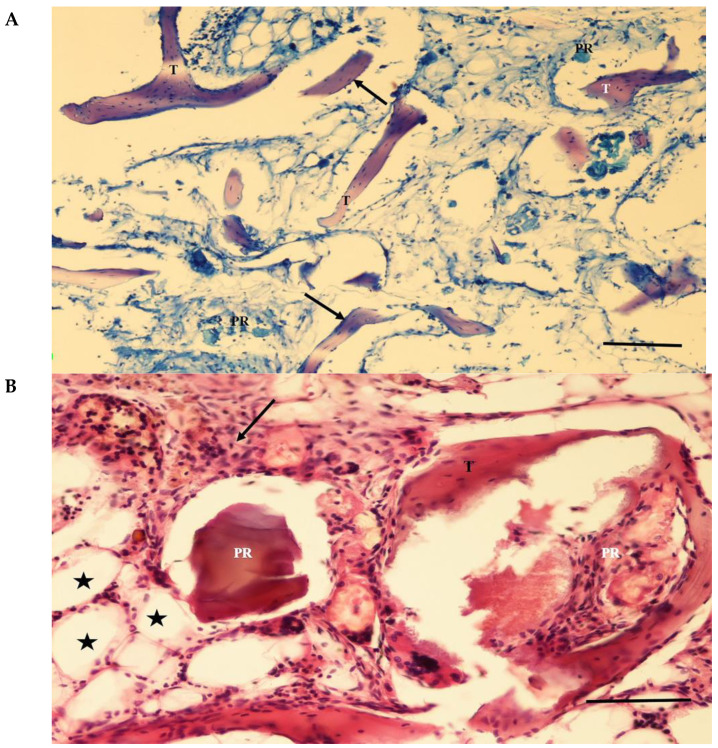
(**A**) A section from the adjacent defect of the MP+ group. The eggshell particles (PR) are highly resorbed. Thin bone trabeculae (T) lined with osteoblastic cells (arrows) surround some of the particles. Pappenheim’s panoptic stain Magnification bar: 100 µm. (**B**) A section from the adjacent defect site of the MP+ group. Thin bone trabeculae (T) surround a highly resorbed eggshell particle (PR). Adipose cells (asterisks) and connective tissue rich in mononuclear cells (arrow) fill between the particle remnants. Intense mononuclear cell infiltration is evident. Fibrous capsule formation surrounding the particles is not seen. Haematoxylene-eosin staining. Magnification bar: 100 µm.

The screw bodies in the cortical region of the MP+ group were covered with tissue similar to that of the PC group. The eggshell particles were highly resorbed in the adjacent socket. Thin bone trabeculae lined with osteoblastic cells surrounded some of the particles. The interarticular region was filled with blood vessels and connective tissue rich in mononuclear cells. Intense mononuclear cell infiltration and a few multinucleated foreign-body giant cells were observed in some areas. Fibrous capsule formation surrounding the particles was not observed (Figure 8).

**Figure 9:**
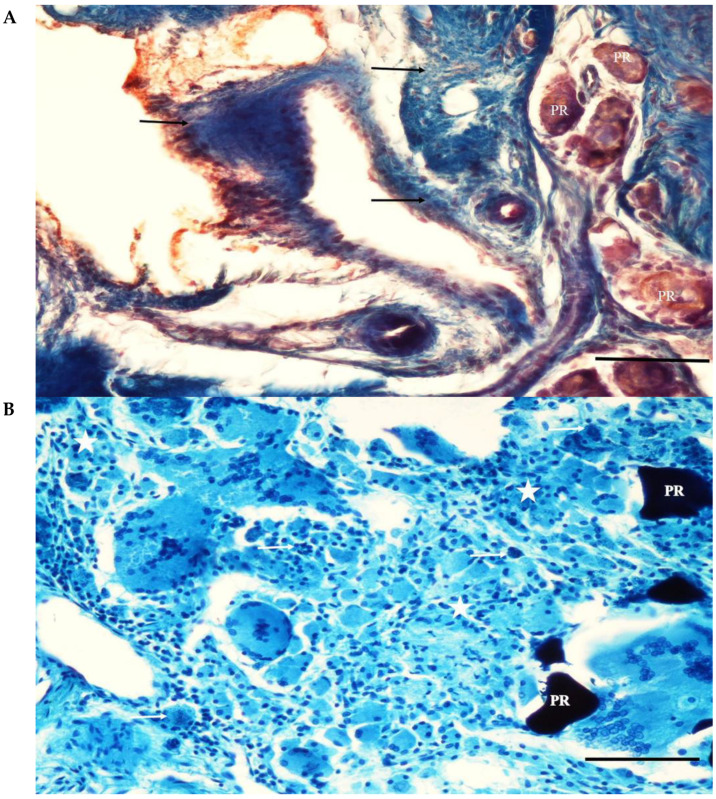
(**A**) Eggshell particles (PR) have resorbed at different levels, with some being large and others being small. The interparticular region is mainly filled with connective tissue and fibrocartilage (arrows). New bone formation is not seen. Trichrome staining. Magnification bar: 100 µm. (**B**) A section through the adjacent defect area of the MP group. The remnants of eggshell particles (PR) and the intervening areas are covered by dense connective tissue (asterisk) filled with mononuclear cells (arrow). Trichrome staining. Magnification bar: 100 µm.

In the MP group, the particles were resorbed at different levels. Osteoblastic cells were seen as simple cuboidal cell lines on the surfaces of the graft particles. Primary bone trabeculae were found on some trabecular surfaces. Intense mononuclear cell infiltration and a few multinucleated foreign-body giant cells were observed in some areas. Fibrous capsule formation surrounding the particles was not observed (Figure 9).

### 3.4. Histomorphometric Results

**Figure 10:**
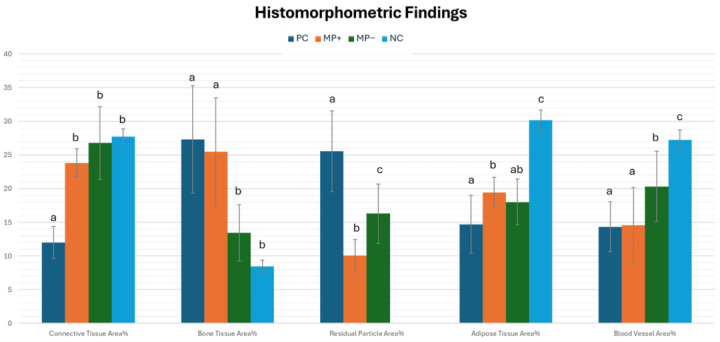
Histomorphometric examination findings. ^a–c^ Different superscripts in each row indicate a statistically significant difference between groups after post hoc analysis.

The highest bone area percentage was observed in the PC group (27.31%) (*p* = 0.00), followed by the MP+ group (25.5%), MP− group (13.44%), and NC group (8.45%). The NC group had the lowest percentage of bone area. Although the NC group had a lower mean bone area percentage than the MP− group, the difference between them was not significant (*p* = 0.365). The particle residue ratio of the PC group was significantly (*p* < 0.05) higher than that of the MP+ and MP− groups. The residual particle area of the MP− (16.3%) group was significantly higher than in the MP+ (10.06) group (*p* = 0.015). The adipose tissue score of the NC group was significantly (*p* = 0.00) higher than that of the other groups. The lowest adipose tissue score was in the PC group (14.7%). The vessel area was highest in the NC group (27.24%) and lowest in the PC group (14.34%). The mean vessel area ratios of the PC and MP+ groups were quite similar (Table 3) (Figure 10).

## 4. Discussion

This experimental study evaluated the efficacy of a new injectable bone substitute containing OE particles with and without eggshell membrane protein. The results were compared with those of a commercially available injectable bone graft comprising biphasic calcium sulfate. As a result of the analysis, our null hypothesis is rejected.

In a previous study, an additional circumferential slot was created in the cervical area for filling with an injectable graft material [23]. However, it should be noted that these studies were primarily performed on the mandibles of animals such as sheep, dogs, and pigs [23,24]. This is the first time this material has been tested on a living body, so we chose the rabbit model for our study. Male New Zealand rabbits were preferred because of their rapid healing time, ease of management, availability, and low cost. In addition, this group’s incidence of health problems is low [18]. The rabbit femur has a medullary cavity with minimal cancellous bone and bone marrow. Our preliminary study showed, in agreement with the literature, that creating a circumferential defect in the neck region of the implants did not result in the primary stability of the implant. To avoid the inadequate healing process that may result from the lack of primary stability of the implants, we created a defect adjacent to the neck region instead of a circumferential defect. This allowed us to study the implant–graft interaction while ensuring the primary stability of the implants. Previous researchers have mostly used implants with a diameter of 8–10 mm in experimental studies in rabbit bone [25]. To address the primary stability issue mentioned earlier, we used 11.5 mm implants in our study and placed them bicortically.

Our study used membranes in all the defect groups to ensure standardization. Resorbable cross-linked collagen membranes were preferred due to the unpredictable complication risk of non-resorbable membranes, the potential risk to the regenerating bone during secondary surgery, and the cost [26]. At the end of week 12, the membranes used in all groups were found to be completely resorbed.

Although autografts and allografts are often the preferred treatments for bone loss, they have several disadvantages. These include limited availability of donor tissue, donor site morbidity, unfavorable immunogenic host response, potential disease transmission, and difficulty in achieving the desired shape [27]. Allografts have additional problems such as increasing material scarcity and supply difficulties due to legal restrictions on tissue donation and new medical device regulations [28]. In contrast, ostrich farming is recognized as a thriving branch of the productive poultry industry in most countries. Production characteristics such as ostrich farm profitability, female fertility, egg production, hatchability, and growth performance make eggshells readily available and cost-effective [29].

Bird eggshells, particularly ostrich eggshells, have been proposed as a potential bone grafting material in maxillofacial surgery [28,29]. The mineralization process of avian eggshells is known for its rapid and intense deposition of calcium salts during biological mineralization [30]. Previous studies have investigated using eggshell powder from various avian species as a bone grafting material [31,32]. OE particles have been tested as a bone substitute in facial and cranial reconstructive surgery in animal models [15,23,26,33]. Man et al. suggested that OE particles could be a valuable filler for limited bone defects in non-weight-bearing areas and that they could be used for bone augmentation. In addition to the role of the inorganic matrix of the eggshell as a tissue scaffold, it also contains many bioactive molecules, mainly type I, V, and X collagens, osteopontin, and sialoproteins, some of which play an active role in the biomineralization and control of this process during shell formation. In addition, organic matrix proteins in eggshells are bioactive molecules that can alter the calcite crystal morphology and precipitation rate during shell formation [34]. The results of previous studies revealed that the particles were osteoproductive and bound to the recipient’s bone and that smaller OE particles were excessively resorbed shortly after the operation [32]. OE particles appeared biocompatible, but a slight infiltration of lymphocytes around the particles was observed 12 months after surgery. The researchers suggested that this reaction may have resulted from a rapid degradation process, releasing more material from smaller particles [35]. In this experiment, no excessive foreign-body reaction characterized by encapsulation, dense PMNL infiltration, necrotic tissue, or numerous giant cells was observed in any of the grafts. Dupoirieux et al. observed a mild inflammatory reaction to eggshell particles after 1 month, which gradually decreased in the following stages [36]. Since previous experiments gave similar results, the OE implant prepared under the present experimental conditions was considered biocompatible [37]. A bone substitute should be easy to shape and handle, and it should retain its initial volume [38]. In this study, the bone cement mixes could be easily applied without side effects. In a recent study, Ünsal et al. coated OE particles with carboxymethylcellulose or gelatin and evaluated their efficacy on new bone formation [33]. In this study, the bone cement was prepared containing micronized OE powder, OE membrane protein, gum arabic, sodium alginate, and CMC. CMC is a safe and inert biopolymer that improves particulate bone grafts’ clinical properties by enhancing particulate composites’ handling properties [20,21]. Although CMC is well tolerated and has osteoconductive properties, in gel form, it can inhibit bone formation [23]. OE membrane protein plays an important role in fibrocyte adhesion. Sodium tri-metaphosphate and sodium alginate contribute to the hardening of bone cement. The resorption of eggshell particles was advanced in both the MP+ and MP− groups. The interparticular regions were filled with fine bone trabeculae, blood vessels, and connective tissue rich in mononuclear cells. Dense mononuclear cell infiltration and a few multinucleated foreign-body giant cells were observed in some areas. However, a fibrous capsule surrounding the particles was not evident. These results showed that the cement material used in the MP+ and MP− groups was biocompatible and osteoproductive, although slight lymphocyte infiltration around the particles was observed. In this study, the PC group showed better healing and ossification scores than the experimentally prepared cement groups. The percentage of bone tissue was highest (*p* < 0.05) in the PC group (27.31%), followed by the MP+ group (25.5%) and the MP− group (13.44%). The lowest percentage was observed for the NC group (8.45%). However, the number of residual particles and the adipose tissue ratios in the PC group were significantly higher than those in the MP+ and MP− groups. Ünsal et al. reported that rapidly resorbing graft particles were replaced by connective and vascular tissue and that new bone tissue formation began in this area. Within months, new bone cells replaced the connective and vascular tissue in the defect area [33].

In theory, BIC is positively correlated with implant stability and interfacial strength, which result from physical contact between the implant and the surrounding alveolar bone [39]. When microtomography results were evaluated, it was observed that the PC group material had the highest BIC value, with a mean value of 37.0105, and it was statistically different from all the other groups (*p* < 0.05). In a recent study, Kang et al. compared the effects of different graft materials on osteointegration in a canine model [24]. The BIC values they found ranged from 55% to 74%. The different results were because the study models and experimental animals were different.

Durmuş et al. strongly stressed that ostrich eggshell as a particulate graft has a remarkable osteoproductive activity and might be a potential graft material. They also suggested that novel eggshell processing, graft preparation, and sterilization methods that preserve the activity of bioactive proteins should be developed for ostrich eggshell particles. The use of this safe and inexpensive material is limited to their use in non-force-bearing areas [35]. In addition, the change in hardening time due to manual mixing is among the issues that need to be improved. The limitations of this study include the need for more data on the early and long periods of bone healing, as the tissue sample was taken at 12 weeks. In addition, the rabbit femur used in this study has different morphological characteristics from the human jaw bone and may behave differently during the healing process. Therefore, studies in other laboratory animals, such as sheep, dogs, or pigs, are needed to obtain results closer to those expected in humans.

## 5. Conclusions

Based on the results of this study, the experimentally produced injectable bone substitute containing OE particles and eggshell membrane protein was found to have biocompatibility and osteoproductive properties, although it is not yet as good as Bond Apatite, a commercial product. These properties suggest that this material may be a promising option for bone tissue engineering and deserves further investigation. However, before this material can be used in clinical applications, further trials are needed in other experimental species such as sheep, dogs, and pigs, alone or in combination with other materials. Such additional studies are essential for us to better understand the safety, efficacy, and potential uses of this material.

## Figures and Tables

**Table 1:** The ingredients of the the eggshell membrane protein-containing (MP+) and non-containing (MP−) graft formulas.

	Gram	
Contents	MP+	MP−
Calcium Phosphate From Ostrich Eggshell Powder	20 g	20 g
Ostrich Eggshell Memrane Protein	0.25 g	-
Calcium Chloride	0.5 g	0.5 g
Sodium Tri-Meta Phosphate	1 g	1 g
Carboxy Methyl Cellulose	0.5 g	0.5 g
Gum Arabic	0.25 g	0.25 g
Alginic Acid	0.25	0.25

**Table 2:** µ-CT scores of the groups.

	PC (*n* = 8)	MP+ (*n* = 8)	MP− (*n* = 8)	NC (*n* = 8)	* *p* Value
BIC %	37.01 ± 3.7 ^a^	29.09 ± 1.4 ^c^	26.4 ± 2.6 ^bc^	24.45 ± 2.0 ^b^	*p* < 0.01

* One-way ANOVA (analysis of variance) test followed by Tukey’s HSD post hoc test for multiple comparisons. ^a–c^ Different superscripts in each row indicate a statistically significant difference between groups after post hoc analysis.

**Table 3:** Histomorphometric examination findings of the groups and their statistical analysis of the data.

Groups (*n* = 8)	Connective Tissue Area%	Bone Tissue Area%	Residual Particle Area%	Adipose Tissue Area%	Blood Vessel Area%
PC	12.00 ± 2.4 ^a^	27.31 ± 8 ^a^	25.53 ± 6.00 ^a^	14.7 ± 4.3 ^a^	14.34 ± 3.7 ^a^
MP+	23.81 ± 2.1 ^b^	25.5 ± 8.00 ^a^	10.06 ± 2.4 ^b^	19.45 ± 2.2 ^b^	14.59 ± 5.6 ^a^
MP−	26.78 ± 5.4 ^b^	13.44 ± 4.2 ^b^	16.3 ± 4.4 ^c^	18.01 ± 3.4 ^ab^	20.32 ± 5.2 ^b^
NC	27.75 ± 1.1 ^b^	8.45 ± 0.9 ^b^	null	30.19 ± 1.5 ^c^	27.24 ± 1.5 ^c^
* *p* values	*p* < 0.01	*p* < 0.01	*p* < 0.01	*p* < 0.01	*p* < 0.01

* One-way ANOVA (analysis of variance) test followed by Tukey’s HSD post hoc test for multiple comparisons. ^a–c^ Different superscripts in each row indicate a statistically significant difference between groups after post hoc analysis.

## Data Availability

The original contributions presented in the study are included in the article, further inquiries can be directed to the corresponding author.
